# The potential of *Bacillus* species isolated from *Cinnamomum camphora* for biofuel production

**DOI:** 10.1186/s12934-024-02402-4

**Published:** 2024-05-15

**Authors:** Noura Sh.A. Hagaggi, Eman A. El Rady

**Affiliations:** 1 Botany Department, Faculty of Science, Aswan University, Aswan, 81528 Egypt; 2 Chemistry Department, Faculty of Science, Aswan University, Aswan, 81528 Egypt

**Keywords:** Endophyte, *Bacillus*, *Cinnamomum camphora*, Alkane, Biofuel, Production

## Abstract

**Background:**

Increasing concerns about climate change and global petroleum supply draw attention to the urgent need for the development of alternative methods to produce fuels. Consequently, the scientific community must devise novel ways to obtain fuels that are both sustainable and eco-friendly. Bacterial alkanes have numerous potential applications in the industry sector. One significant application is biofuel production, where bacterial alkanes can serve as a sustainable eco-friendly alternative to fossil fuels. This study represents the first report on the production of alkanes by endophytic bacteria.

**Results:**

In this study, three Bacillus species, namely *Bacillus atrophaeus* Camph.1 (OR343176.1), *Bacillus spizizenii* Camph.2 (OR343177.1), and *Bacillus aerophilus* Camph.3 (OR343178.1), were isolated from the leaves of *C. camphora*. The isolates were then screened to determine their ability to produce alkanes in different culture media including nutrient broth (NB), Luria–Bertani (LB) broth, and tryptic soy broth (TSB). Depending on the bacterial isolate and the culture media used, different profiles of alkanes ranging from C_8_ to C_31_ were detected.

**Conclusions:**

The endophytic *B. atrophaeus* Camph.1 (OR343176.1), *B. spizizenii* Camph.2 (OR343177.1), and *B. aerophilus* Camph.3 (OR343178.1), associated with *C. camphora* leaves, represent new eco-friendly approaches for biofuel production, aiming towards a sustainable future. Further research is needed to optimize the fermentation process and scale up alkane production by these bacterial isolates.

## Background

Fossil fuels have been used for decades to produce liquid fuels such as diesel, gasoline, and kerosene. However, it is predicted that petroleum reserves will be depleted within 40 years [1]. This has raised concerns about the global petroleum supply, and environmental issues such as global warming and climate change. As a result, there is growing interest in exploring alternative fuel sources [2]. Consequently, a significant focus has been on developing alternative biosynthesis methods for sustainable and eco-friendly biofuel.

Alkanes are hydrocarbons that are essential for biofuel production. They are the key building blocks of renewable biodiesel. Alkanes offer a sustainable alternative to fossil fuels, aligning with global efforts to reduce climate change and improve environmental sustainability [3]. The stable chemical structure of alkanes also helps biofuels retain their quality and performance in long-term storage. This stability is critical for meeting the requirements of individual consumers as well as commercial and industrial sectors [4]. The traditional commercial production of alkanes constantly increases production costs, non-renewable energy consumption, and gaseous pollutants [5]. The microbial biosynthesis of alkanes can be a promising sustainable alternative for chemical production [6]. Incorporating microorganisms into the global future of green energy can achieve a distributed and sustainable supply chain that is safe, reliable, and responsive to ever-changing global demand [7, 8].

The biosynthesis of alkanes by bacteria has attracted significant attention in recent years due to its potential for biofuel production [9]. Bacterial alkanes possess desirable properties, including high energy content and low freezing points compared to other biofuel sources, making them well-suited for specific applications in the aviation or automotive industries [10].

*Cinnamomum camphora* (L.) J. Presl., popularly known as the camphor tree, is a member of the Lauraceae family. It is native to China, Korea, and Japan and is extensively cultivated in Asia, Africa, North America, and Australia [11]. Every part of the plant contains volatile organic compounds that have medicinal properties [12]. Previous studies have shown that it is possible to utilize endophytes that inhabit plant tissues to synthesize compounds similar to those produced naturally by the host plants [13]. This approach avoids the risk of over-harvesting or the negative effects of climate change on plants, which can affect the production of these compounds [14]. Although alkane biosynthesis has been recognized in various microorganisms, including cyanobacteria, genetically modified bacteria, yeasts, and fungi, no studies have reported the production of alkanes by endophytic bacteria [15]. Therefore, this study aims to isolate endophytic bacteria from *C. camphora* leaves and screen the production of alkanes by the isolates in different culture media. The study is an attempt to find renewable sources for bio-alkanes that may be promising for sustainable biofuel production.

## Materials and methods

### Plant material

Leaves from healthy trees of *C. camphora* were collected from Aswan City, Egypt (24° 5′ 20.1768'' N, 32° 53′ 59.3880'' E) and brought directly to the Aswan University bacteriology laboratory for the isolation of endophytic bacteria.

### Isolation and identification of endophytic bacteria

The surfaces of the leaves were sterilized using 5% NaClO, 70% CH_3_CH_2_OH, and autoclaved distilled water, respectively [16]. In a 9 mL sterile saline solution, 1 g of the leaves was mashed well. One milliliter of the resulting suspension was then inoculated in trypticase soy and nutrient agar plates. Plates were incubated for 72 h at 37 ℃. Three isolates coded as Camph.1, Camph.2, and Camph.3 were subjected to molecular identification by partially sequencing their 16S rRNA genes. The amplification primers 27F and 1492R were used [17]. The separation of PCR products was performed using 1% (w/v) agarose gel. The sequencing of the obtained bands was commercially performed at SolGent Co., Korea. The sequence similarity and identity percentages were determined using the NCBI website (https://www.ncbi.nlm.nih.gov/). An accession number was gained for each isolate after submitting its 16S rRNA gene partial sequence into the NCBI database. The phylogenetic relationship among the present isolates and the other close members of NCBI was constructed using neighbor-joining analysis in MEGA X 10.1.7 software [18].

### Determination of bacterial growth curves

The growth curves of the bacterial isolates grown in nutrient broth (NB), Luria–Bertani (LB) broth, and tryptic soy broth (TSB) were determined using the turbidimetric method [19]. In 250 mL conical flasks, 50 mL of each medium was prepared and autoclaved. The flasks were inoculated with 100 µL of each bacterial inoculum (1.5 × 10^8^ CFU/mL, OD_600_ = 0.1). The flasks were incubated at 37 °C under shaking (150 rpm). The optical density was read at 600 nm at intervals of 10 h until the stationary phase was reached. The flasks without bacterial inoculums served as controls. The experiment was conducted three times.

### Fermentation conditions

The freshly prepared inoculum (100 µL of 1 × 10^7^ CFU/mL) of each bacterial isolate was inoculated in 500 mL flasks containing 100 mL of three different broth culture media: NB, LB, and TSB. The flasks were incubated for 48 h at 37 °C and 150 rpm. Flasks containing media without bacterial inoculum were used as controls. Triplicates were made for all fermentations.

### Extraction and GC/MS analysis of alkanes

Hexane was added to the bacterial cultures in a ratio of 1:1 (v/v) and homogenized well. The solvent layers were then separated and concentrated using a rotary evaporator at 40 °C and 130 rpm under pressure. Each extract (1 mg) was redissolved in 10 mL of hexane. The GC/MS system used in this study was the Agilent Technologies 7890A GC/5977A MSD supplied with a TR-5MS GC column (30 m, 0.25 mm ID, and 0.25 μm film). The sample (1 μL) was injected into the column, and the oven temperature was initially set at 30 °C for 1 min. The temperature was then increased at a rate of 10 °C/min until reaching 200 °C, where it was held for an additional 1 min. The carrier gas, helium, was used at a flow rate of 20 mL/min. The retention times of the sample peaks were compared with NIST11.L standard reference compounds. The alkane standard mixture (C_7_-C_40_, Millipore Sigma^™^ Supelco^™^) was used to quantify the alkanes in the samples.

### Effect of carbon sources on alkane production

In conical flasks, the basal medium consisted of the following components per liter: KH_2_PO_4_ (1.3 g), MgSO_4_.7H_2_O (0.2 g), NaCl (5 g), (NH_4_)_2_SO_4_ (1 g), and yeast extract (5 g) was supplemented with different carbon sources including glucose, sucrose, and sugar cane molasses, each at a concentration of 10 g/L. Flasks were then inoculated with 100 µL of a freshly prepared inoculum containing 1 × 10^7^ CFU/mL. Flasks were incubated for 48 h at 37 °C and 150 rpm. Flasks containing media without bacterial inoculum were used as controls. Triplicates were prepared for all fermentations. Alkanes were extracted and analyzed using the method described above.

## Results and discussion

The use of biofuels has become crucial in addressing the worldwide concerns of the energy crisis and climate change. Microbial alkanes provide a renewable, eco-friendly, and promising source for the sustainable production of biofuels [20]. Unlike fossil fuels, biofuels derived from microbial alkanes not only decrease carbon emissions but also mitigate the effects of global warming [21]. The low toxicity and biodegradability of bacterial alkanes make them eco-friendly alternatives to synthetic alkanes, serving various applications [22].

### Isolation and identification of endophytic bacteria

**Fig.1:**
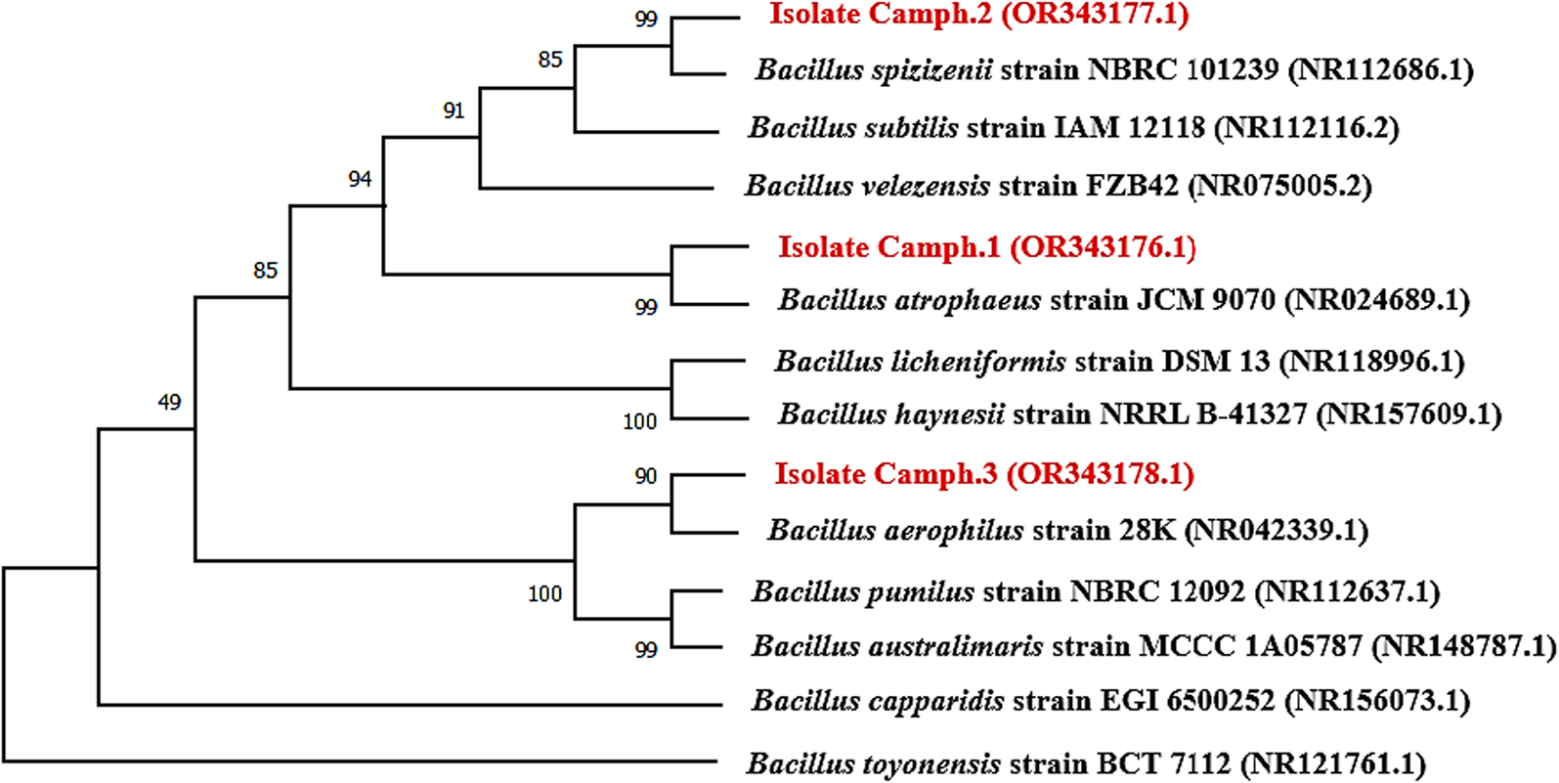
The phylogenetic relationship among the isolates Camph.1, Camph.2, Camph.3, and the closely related species from the NCBI database using the neighbor-joining method in MEGA X10.1.7 software

In this study, three endophytic bacteria were isolated from the leaves of *C. camphora* and coded as Camph.1, Camph.2, and Camph.3*.* Based on 16S rRNA gene sequence analysis, the isolates Camph.1, Camph.2, and Camph.3 were found to be quite similar to *Bacillus atrophaeus* (NR024689.1), *Bacillus spizizenii* (NR112686.1), and *Bacillus aerophilus* (NR042339.1), respectively (Fig. 1). The NCBI accession numbers of the isolates Camph.1, Camph.2, and Camph.3 are OR343176.1, OR343177.1, and OR343178.1, respectively. It was observed that the genus *Bacillus* was dominant among endophytes, this may be attributed to the ability of *Bacillus* spp. to form spores and tolerate extreme temperatures in the Aswan region. This finding agreed with previous studies which reported the isolation of *Bacillus* spp. from different plants grown in Aswan [23–25].

### Determination of bacterial growth curves

**Fig. 2:**
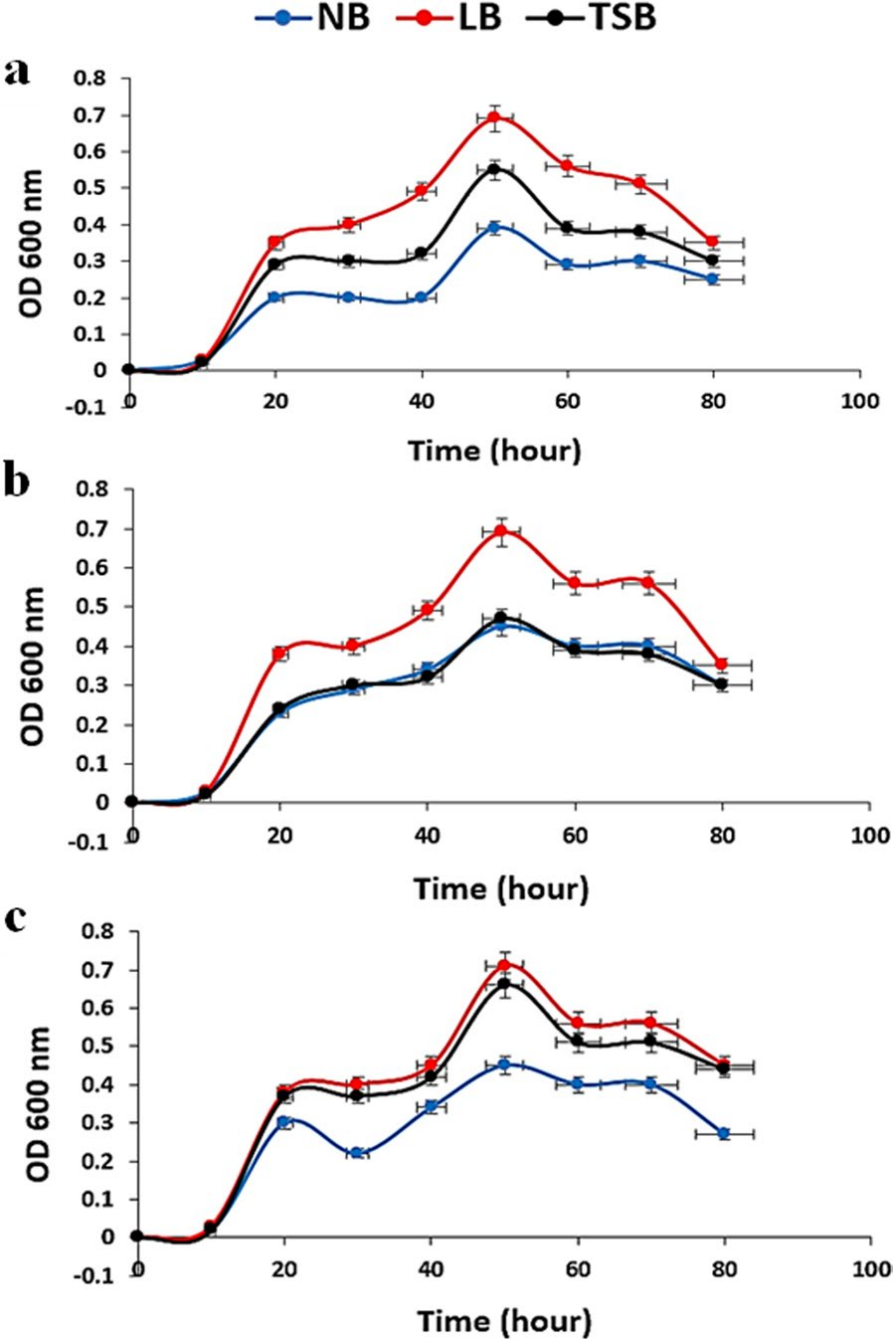
The bacterial growth curves for *B. atrophaeus* Camph.1 (**a**), *B. spizizenii* Camph.2 (**b**), and *B. aerophilus* Camph.3 (**c**), in NB, LB, and TSB media. The bars represent the standard errors of the means

From a commercial perspective, the growth of microorganisms is a significant challenge in the industrial production of valuable chemicals [26]. Therefore, the growth curve for each bacterial isolate was determined in each culture medium. It was observed that the exponential phase of the three isolates began after 20 h of incubation and extended until 50 h. The stationary phase continued for 20 h, after which the growth rate declined (Fig. 2). Generally, the growth rate was higher in LB followed by TSB. The NB, on the other hand, had the lowest growth rate for all isolates (Fig. 2).

### GC/MS analysis of alkanes

The production of alkanes was detected by GC/MS analysis after growing the bacterial isolates in three different culture media: NB, LB, and TSB. It was interesting to note that the alkane profiles vary depending on the growth medium and the bacterial strain. For *B. atrophaeus* Camph.1, the major number of alkanes was detected in the LB medium, where fourteen alkanes were evaluated, including Heptane-2,2,4,6,6-pentamethyl, Decane-2,4,6-trimethyl, Octadecane-1-iodo, Tetradecane, Tridecane-3-methyl, 10-Methylnonadecane, Hexacosane, Tetracosane, Eicosane-2-methyl, Undecane-2,9-dimethyl, Heptadecane-9-octyl, Heptadecane-2-methyl, Nonadecane-2-methyl, and 2-methyloctacosane. TSB medium contained ten alkanes, which were Nonane-2,2,3-trimethyl, Octane-2-methyl, Tetradecane-4-ethyl, Decane-3-methyl, Heptadecane-2-methyl, Pentacosane, Octadecane, Hexacosane, Cyclobutane-1,2-diethyl, and Eicosane. NA medium contained seven alkanes, which included Heptane-2,2,4,6,6-pentamethyl, Decane-2,4,6-trimethyl, Hexadecane-3-methyl, Hexacosane, Octadecane-1-iodo, 1,3,5,7,9-Pentaethyl-1,9-dibutoxypentasiloxane, and Hentriacontane.

Fourteen alkanes were produced by *B. spizizenii* Camph.2 in LB medium (Table 5). In comparison, eleven alkanes were detected in both NB and TSB (Tables 4 and 6) and (Fig. 4). LB medium included Heptane-2,2,4,6,6-pentamethyl, Undecane-3,9-dimethyl, Decane-3,8-dimethyl, Tridecane-1-iodo, Hexadecane-2,6,11,15-tetramethyl, Pentacosane, Octadecane, Decane-3-methyl, Hexadecane, Hentriacontane, Eicosane, Heneicosane, Heptacosane, and 2-Bromo dodecane (Table 5). NB medium contained Nonane-2,2,3-trimethyl, Dodecane, Eicosane, Hexadecane, 2,2-Dimethyleicosane, Octacosane, Heptadecane-2-methyl, Hexadecane-8-hexyl-8-pentyl, 5-Ethyl-5-methylnonadecane, Cyclobutane-1,2-diethyl-trans, and Octane-2,5,6-trimethyl (Table 4). On the other hand, TSB medium included Heptane-2,2,4,6,6-pentamethyl, Undecane-4,7-dimethyl, Hexadecane, Heneicosane, Hexacosane, Hentriacontane, Heptadecane-2-methyl, Heptadecane-9-octyl, Octacosane, Octadecane-1-iodo, and Pentadecane-2-methyl and (Table 6).

**Fig. 3:**
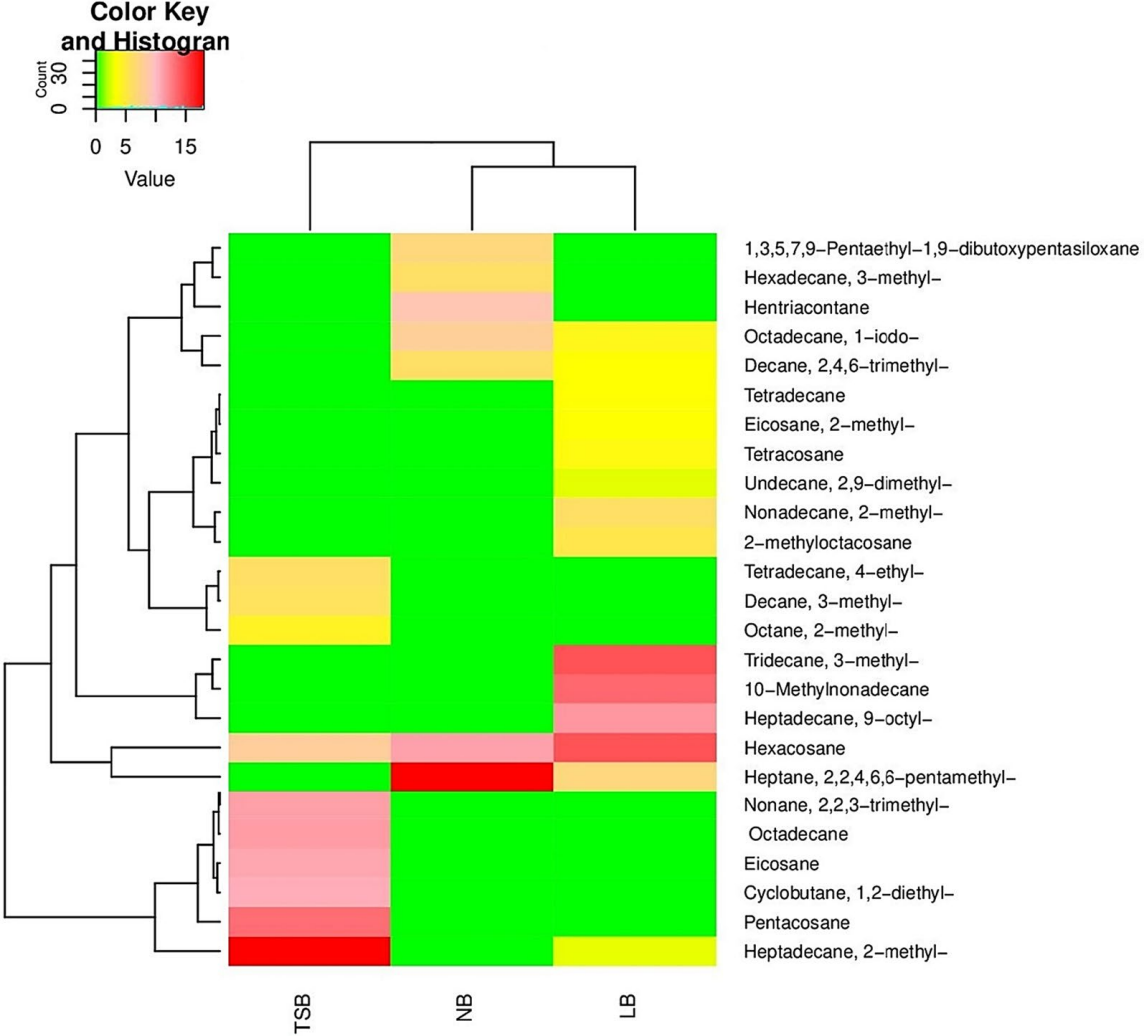
The heatmap displays the amounts of bio-alkanes (mg alkane/L culture) produced by *B. atrophaeus* Camph.1 in NB, LB, and TSB media

**Fig. 4:**
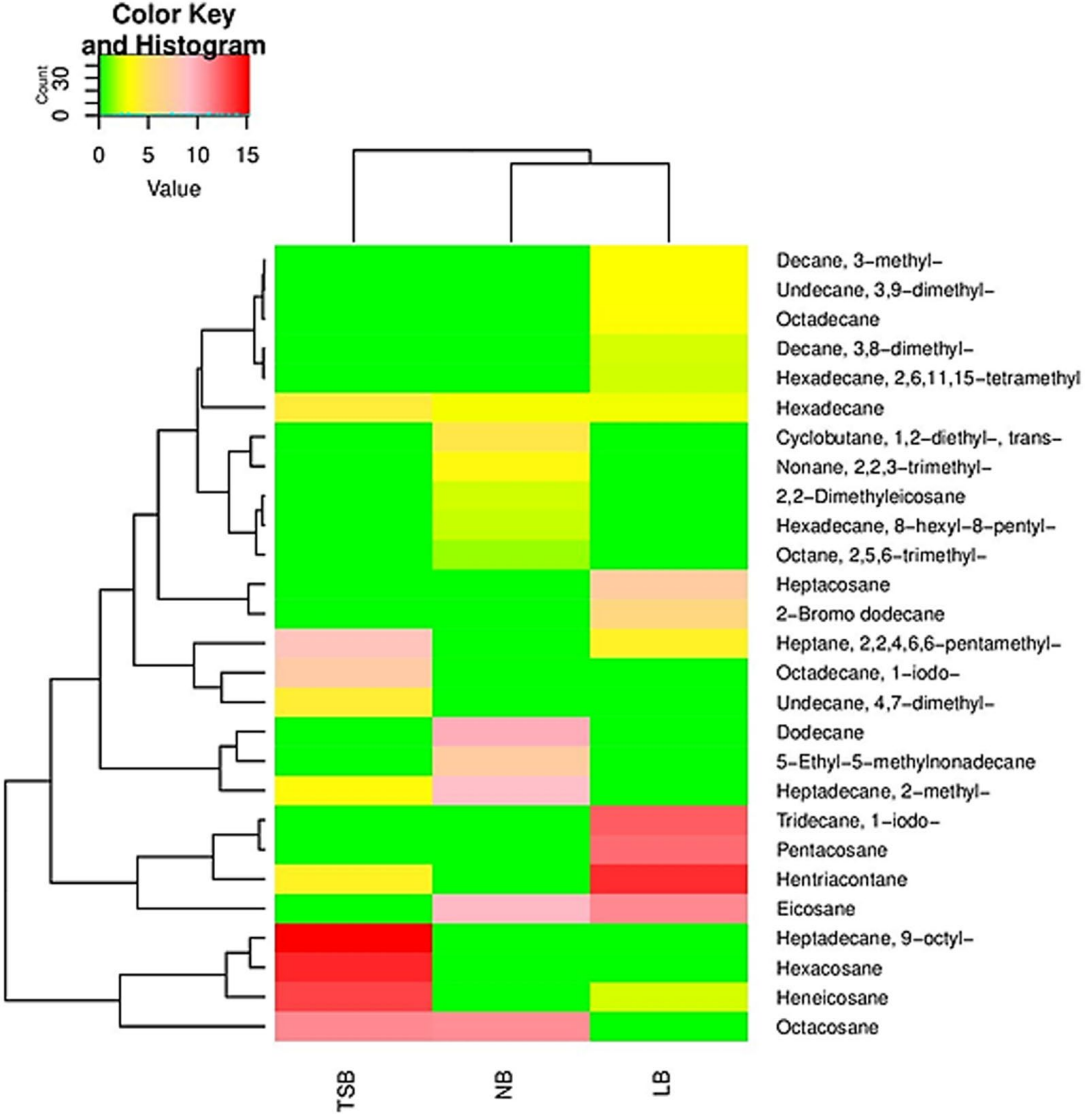
The heatmap displays the amounts of bio-alkanes (mg alkane/L culture) produced by *B. spizizenii* Camph.2 in NB, LB, and TSB media

**Fig. 5:**
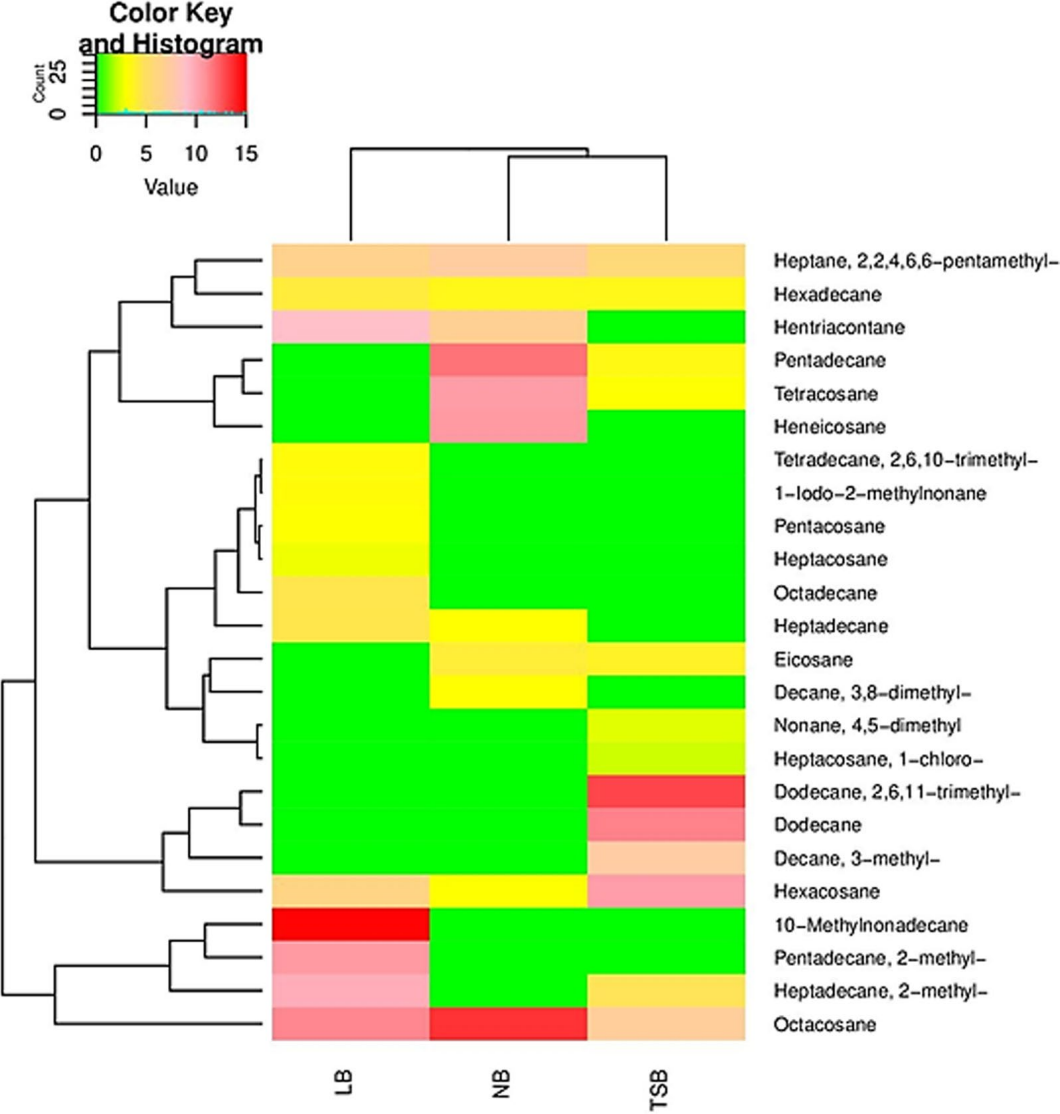
The heatmap displays the amounts of bio-alkanes (mg alkane/L culture) produced by *B. aerophilus* Camph.3 in NB, LB, and TSB media

On the other hand, *B. aerophilus* Camph.3 produced eleven alkanes when grown in NB medium: Heptane-2,2,4,6,6-pentamethyl, Decane-3,8-dimethyl, Eicosane, Hexacosane, Pentadecane, Tetracosane, Hexadecane, Heptadecane, Heneicosane, Hentriacontane, and Octacosane (Table 7 and Fig. 5). Fourteen alkanes were produced in both LB and TSB media: Heptane, 2,2,4,6,6-pentamethyl, 1-Iodo-2-methylnonane, Hexadecane, Tetradecane-2,6,10-trimethyl, 10-Methylnonadecane, Octacosane, Pentacosane, Heptacosane, Heptadecane, Heptadecane-2-methyl, Hentriacontane, Octadecane, Pentadecane-2-methyl, and Hexacosane (Table 8 and Fig. 4) and Heptane-2,2,4,6,6-pentamethyl, Nonane-4,5-dimethyl, Heptadecane-2-methyl, Eicosane, Dodecane-2,6,11-trimethyl, Heptacosane-1-chloro, Dodecane, Tetracosane, Hexadecane, Pentadecane, Hexacosane, Octacosane, Decane-3-methyl, and Decane-4-methylene (Table 9 and Fig. 5), respectively.

Interestingly, the profiles of alkanes released by the three bacterial isolates in the three tested culture media differed. For all isolates, the highest number of alkanes was detected in the LB medium (Fig. 3, 4,5). This finding aligns with previous studies that have reported a significant effect of medium composition on the profiles of volatile organic compounds released by microorganisms [27].

### Effect of carbon sources on alkane production

Interestingly, various alkanes were produced by the three bacterial isolates using glucose, sucrose, and sugar cane molasses as carbon sources. This is consistent with previous studies that reported significant differences in hydrocarbon profiles produced by microorganisms based on carbon sources [28]. *B. atrophaeus* Camph.1 produced fifteen different alkanes using glucose as a carbon source which are Tetradecane, 2,2-dimethy (3.8 mg/L), Undecane, 2-methyl (2.38 mg/L), Eicosane (12.47 mg/L), Decane, 3,8-dimethyl (2.29 mg/L), Heptadecane, 4-methyl (12.03 mg/L), Hentriacontane (3.2 mg/L), Octadecane, 2-methyl (3.1 mg/L), Heneicosane (2.9 mg/L), Hexadecane (2.8 mg/L), Hexacosane (13.8 mg/L), Hexadecane, 2,6,10,14-tetramethyl (2.6 mg/L), Octacosane (7.39 mg/L), Pentacosane (6.08 mg/L), 2-methyloctacosane (2.15 mg/L), and Heneicosane, 3-methyl (2.85 mg/L). On the other hand, the GC/MS analysis revealed a total of twelve alkanes produced by *B. atrophaeus* Camph.1 when grown in a medium supplemented with sucrose, which were Heptane, 2,2,4,6,6-pentamethyl (8.36 mg/L), Undecane, 3,7-dimethyl (4.08 mg/L), Decane, 2,9-dimethyl (4.15 mg/L), Decane, 2-methyl (13.19 mg/L), Heptadecane, 8-methyl (14.02 mg/L), Nonadecane, 3-methyl (3.74 mg/L), Hentriacontane (3.25 mg/L), Hexadecane (3.49 mg/L), Heneicosane (15.21 mg/L), 2-methyloctacosane (11.18 mg/L), Octacosane (7.57 mg/L), and Octadecane, 1-iodo (7.22 mg/L). Ten alkanes were detected in a medium supplemented with sugar cane molasses including Decane, 2,2,3-trimethyl (3.35 mg/L), Octadecane (2.76 mg/L), Heptadecane, 2-methyl (9.69 mg/L), Dodecane (9.23 mg/L), Eicosane (2.46 mg/L), Hexadecane (2.86 mg/L), Hexacosane (11.02 mg/L), Nonane, 4,5-dimethyl (2.19 mg/L), Heneicosane (8.96 mg/L), and 2,2-Dimethyleicosane (1.57 mg/L).

For *B. spizizenii* Camph.2, thirteen alkanes were produced in a glucose-based medium, which are Heptane, 2,2,4,6,6-pentamethyl (6.63 mg/L), Undecane, 4,7-dimethyl (3.18 mg/L), Hexadecane (4.19 mg/L), Decane, 2-methyl (3.26 mg/L), Tridecane, 1-iodo (14.82 mg/L), Heptadecane, 8-methyl (11.21 mg/L), 10-Methylnonadecane (2.98 mg/L), Pentadecane (4.54 mg/L), Octacosane (9.71 mg/L), Hentriacontane (8.95 mg/L), Octadecane (4.55 mg/L), Hexadecane, 2-methyl (10.52 mg/L), and Pentadecane, 3-methyl (6.25 mg/L). Ten alkanes were detected in a sucrose-based medium, including Heptane, 2,2,4,6,6-pentamethyl (7.25 mg/L), Decane, 3,6-dimethyl (3.04 mg/L), Octane, 2,4,6-trimethyl (4.13 mg/L), Pentacosane (2.98 mg/L), Heneicosane (11.80 mg/L), Tridecane, 1-iodo (10.44 mg/L), Hexadecane (3.49 mg/L), Eicosane (3.05 mg/L), Hexacosane (6.85 mg/L), and 2-methyloctacosane (13.55 mg/L). On the other hand, 2,2,7,7-Tetramethyloctane (5.81 mg/L), Decane, 3-methyl (2.56 mg/L), Undecane, 3-methyl (4.75 mg/L), Octadecane, 2-methyl (12.99 mg/L), Hexacosane (11.38 mg/L), Eicosane (2.97 mg/L), Heptadecane (3.53 mg/L), 2-Bromo dodecane (10.37 mg/L), Triacontane (7.06 mg/L), Heneicosane (7.48 mg/L), were produced in sugar cane molasses-based medium by *B. spizizenii* Camph.2.

Seven alkanes including Heptane, 2,2,4,6,6-pentamethyl (17.92 mg/L), Decane, 3,6-dimethyl (6.01 mg/L), Heptacosane (6.05 mg/L), Tetracosane (11.25 mg/L), Pentacosane (8.02 mg/L), Heptadecane, 2-methyl (9.11 mg/L), and Eicosane (5.75 mg/L) were produced in glucose-based medium by *B. aerophilus* Camph.3. Moreover, Decane, 2,2,3-trimethyl (11.32 mg/L), Undecane, 5-methyl (4.37 mg/L), Eicosane (5.98 mg/L), Nonane, 4,5-dimethyl (5.78 mg/L), Heptadecane, 8-methyl (17.89 mg/L), Octacosane (13.33 mg/L), Octadecane (11.47 mg/L), and Octane, 2-methyl (11.01 mg/L) were detected in sucrose-based medium. Sugar cane molasses-based medium achieved the production of twelve alkanes by *B. aerophilus* Camph.3 which are Heptane, 2,2,4,6,6-pentamethyl (6.98 mg/L), Hexacosane (3.36 mg/L), Nonane, 4,5-dimethyl (4.09 mg/L), Decane, 3-methyl (3.38 mg/L), Heneicosane (14.51 mg/L), 10-Methylnonadecane (2.88 mg/L), Docosane (14.53 mg/L), Octadecane (3.77 mg/L), Octadecane, 1-iodo (13.61 mg/L), Hentriacontane (2.78 mg/L), 2-methyloctacosane (11.65 mg/L), and Heptadecane, 9-octyl (5.96 mg/L).

As stated above, the chain length of alkanes produced by the present isolates ranged from C_8_ to C_31_ (Tables 1, 2, 3, 4, 5, 6, 7, 8, 9). Previous studies have reported that bacterial alkanes typically have chain lengths ranging from C_10_ to C_36_, although this can vary depending on the bacterial strain and environmental conditions [29]. The biosynthesis of n-alkanes by various bacteria including *Desulfovibrio* sp., *Clostridium* sp., *Pseudomonas fluorescens*, *Vibrio furnissii* M1, and Engineered *Escherichia coli* has been reported [30–33]. Although endophytic bacteria were known within the biotechnology field for their ability to produce a great variety of sustainable safe, eco-friendly products, there are no reports about their ability to produce alkanes [34]. Therefore, this study is the first documentation of alkane production by endophytic bacteria.

**Table 1:** Bio-alkanes produced by *B. atrophaeus* Camph.1 in NB medium

Alkane name	Structure	Formula	Molecular weight	Retention time	Peak area	mg alkane/L culture
Heptane, 2,2,4,6,6-pentamethyl-		C_12_H_26_	170	4.432	1,863,723	17.923
Decane, 2,4,6-trimethyl-		C_13_H_28_	184	5.215	625,432	6.015
Hexadecane, 3-methyl-		C_17_H_36_	240	7.378	629,978	6.058
Hexacosane		C_26_H_54_	366	9.089	1,170,008	11.252
Octadecane, 1-iodo-		C_18_H_37_I	380	9.495	834,492	8.025
1,3,5,7,9-Pentaethyl-1,9-dibutoxypentasiloxane		C_18_H_48_O_6_Si_5_	500	10.789	722,371	6.947
Hentriacontane		C_31_H_64_	436	11.378	947,253	9.110

**Table 2:** Bio-alkanes produced by *B. atrophaeus* Camph.1 in LB medium

Alkane name	Structure	Formula	Molecular weight	Retention time	Peak area	mg alkane/L culture
Heptane, 2,2,4,6,6-pentamethyl-		C_12_H_26_	170	4.426	1,316,286	6.984
Decane, 2,4,6-trimethyl-		C_13_H_28_	184	5.215	634,388	3.366
Octadecane, 1-iodo-		C_18_H_37_I	380	7.378	772,529	4.099
Tetradecane		C_14_H_30_	198	7.733	638,078	3.386
Tridecane, 3-methyl-		C_14_H_30_	198	9.089	2,736,279	14.519
10-Methylnonadecane		C_20_H_42_	282	11.378	2,565,585	13.613
Hexacosane		C_26_H_54_	366	9.495	2,738,304	14.530
Tetracosane		C_24_H_50_	338	9.587	710,911	3.772
Eicosane, 2-methyl-		C_21_H_44_	296	9.684	624,411	3.313
Undecane, 2,9-dimethyl-		C_13_H_28_	184	11.487	524,687	2.784
Heptadecane, 9-octyl-		C_25_H_52_	352	11.939	2,196,338	11.654
Heptadecane, 2-methyl-		C_18_H_38_	254	9.169	543,692	2.885
Nonadecane, 2-methyl-		C_20_H_42_	282	14.531	1,124,351	5.966
2-methyloctacosane		C_29_H_60_	408	15.240	1,008,527	5.351

**Table 3:** Bio-alkanes produced by *B. atrophaeus* Camph.1 in TSB medium

Alkane name	Structure	Formula	Molecular weight	Retention time	Peak area	mg alkane/L culture
Nonane, 2,2,3-trimethyl-		C_12_H_26_	170	4.426	1,709,131	11.321
Octane, 2-methyl-		C_9_H_20_	128	5.215	661,119	4.379
Tetradecane, 4-ethyl-		C_16_H_34_	226	7.378	903,832	5.987
Decane, 3-methyl-		C_11_H_24_	156	7.739	873,751	5.787
Heptadecane, 2-methyl-		C_18_H_38_	254	9.089	2,701,569	17.894
Pentacosane		C_25_H_52_	352	9.495	2,013,320	13.336
Octadecane		C_18_H_38_	254	11.378	1,731,725	11.470
Hexacosane		C_26_H_54_	366	11.939	1,224,642	8.112
Cyclobutane, 1,2-diethyl-		C_8_H_16_	112	14.531	1,615,566	10.701
Eicosane		C_20_H_42_	282	15.240	1,662,776	11.014

**Table 4:** Bio-alkanes produced by *B. spizizenii* Camph.2 in NB medium

Alkane name	Structure	Formula	Molecular weight	Retention time	Peak area	mg alkane/L culture
Nonane, 2,2,3-trimethyl-		C_12_H_26_	170	4.420	1,262,471	3.354
Dodecane		C_12_H_26_	170	9.089	3,648,878	9.694
Eicosane		C_20_H_42_	282	9.495	3,475,442	9.233
Hexadecane		C_16_H_34_	226	10.062	1,079,883	2.869
2,2-Dimethyleicosane		C_22_H_46_	310	11.206	855,681	2.273
Octacosane		C_28_H_58_	394	11.378	4,149,590	11.024
Heptadecane, 2-methyl-		C_18_H_38_	254	11.939	3,373,007	8.961
Hexadecane, 8-hexyl-8-pentyl-		C_27_H_56_	380	12.059	789,803	2.098
5-Ethyl-5-methylnonadecane		C_22_H_46_	310	14.531	2,794,080	7.423
Cyclobutane, 1,2-diethyl-, trans-		C_8_H_16_	112	14.571	1,681,555	4.467
Octane, 2,5,6-trimethyl-		C_11_H_24_	156	19.091	594,154	1.579

**Table 5:** Bio-alkanes produced by *B. spizizenii* Camph.2 in LB medium

Alkane name	Structure	Formula	Molecular weight	Retention time	Peak area	mg alkane/L culture
Heptane, 2,2,4,6,6-pentamethyl-		C_12_H_26_	170	4.420	1,516,557	3.802
Undecane, 3,9-dimethyl-		C_13_H_28_	184	7.373	1,186,604	2.975
Decane, 3,8-dimethyl-		C_12_H_26_	170	7.733	952,014	2.387
Tridecane, 1-iodo-		C_13_H_27_I	310	9.089	4,977,726	12.479
Hexadecane, 2,6,11,15-tetramethyl		C_20_H_42_	282	9.170	913,946	2.291
Pentacosane		C_25_H_52_	352	9.496	4,801,726	12.038
Octadecane		C_18_H_38_	254	9.685	1,243,122	3.116
Decane, 3-methyl-		C_11_H_24_	156	9.810	1,196,272	2.999
Hexadecane		C_16_H_34_	226	10.062	1,118,006	2.803
Hentriacontane		C_31_H_64_	436	11.378	5,539,119	13.886
Eicosane		C_20_H_42_	282	11.939	4,466,188	11.196
Heneicosane		C_21_H_44_	296	12.029	938,724	2.353
Heptacosane		C_27_H_56_	380	14.531	2,948,890	7.393
2-Bromo dodecane		C_12_H_25_Br	248	15.235	2,428,083	6.087

**Table 6:** Bio-alkanes produced by *B. spizizenii* Camph.2 in TSB medium

Alkane name	Structure	Formula	Molecular weight	Retention time	Peak area	mg alkane/L culture
Heptane, 2,2,4,6,6-pentamethyl-		C_12_H_26_	170	4.426	1,354,335	8.360
Undecane, 4,7-dimethyl-		C_13_H_28_	184	5.215	662,187	4.088
Hexadecane		C_16_H_34_	226	7.373	672,525	4.152
Heneicosane		C_21_H_44_	296	9.089	2,137,083	13.192
Hexacosane		C_26_H_54_	366	9.495	2,272,655	14.029
Hentriacontane		C_31_H_64_	436	9.593	606,217	3.742
Heptadecane, 2-methyl-		C_18_H_38_	254	9.684	527,376	3.256
Heptadecane, 9-octyl-		C_25_H_52_	352	11.378	2,465,248	15.218
Octacosane		C_28_H_58_	394	11.939	1,812,518	11.189
Octadecane, 1-iodo-		C_18_H_37_I	380	14.531	1,227,028	7.575
Pentadecane, 2-methyl-		C_16_H_34_	226	15.235	1,170,809	7.228

**Table 7:** Bio-alkanes produced by *B. aerophilus* Camph.3 in NB medium

Alkane name	Structure	Formula	Molecular weight	Retention time	Peak area	mg alkane/L culture
Heptane, 2,2,4,6,6-pentamethyl-		C_12_H_26_	170	4.432	1,397,729	7.257
Decane, 3,8-dimethyl-		C_12_H_26_	170	5.216	586,155	3.043
Eicosane		C_20_H_42_	282	7.379	796,408	4.135
Hexacosane		C_26_H_54_	366	7.733	574,953	2.985
Pentadecane		C_15_H_32_	212	9.089	2,274,449	11.808
Tetracosane		C_24_H_50_	338	9.496	2,011,445	10.443
Hexadecane		C_16_H_34_	226	10.062	673,346	3.496
Heptadecane		C_17_H_36_	240	11.207	588,982	3.058
Heneicosane		C_21_H_44_	296	11.378	2,036,079	10.571
Hentriacontane		C_31_H_64_	436	11.939	1,319,359	6.850
Octacosane		C_28_H_58_	394	14.531	2,611,310	13.557

**Table 8:** Bio-alkanes produced by *B. aerophilus* Camph.3 in LB medium

Alkane name	Structure	Formula	Molecular weight	Retention time	Peak area	mg alkane/L culture
Heptane, 2,2,4,6,6-pentamethyl-		C_12_H_26_	170	4.432	1,226,975	6.634
1-Iodo-2-methylnonane		C_10_H_21_I	268	5.215	588,941	3.184
Hexadecane		C_16_H_34_	226	7.378	776,012	4.196
Tetradecane, 2,6,10-trimethyl-		C_17_H_36_	240	7.739	603,654	3.264
10-Methylnonadecane		C_20_H_42_	282	9.089	2,740,948	14.820
Octacosane		C_28_H_58_	394	9.495	2,074,784	11.218
Pentacosane		C_25_H_52_	352	9.587	552,547	2.988
Heptacosane		C_27_H_56_	380	9.684	515,221	2.786
Heptadecane		C_17_H_36_	240	11.201	840,206	4.543
Heptadecane, 2-methyl-		C_18_H_38_	254	11.378	1,796,476	9.714
Hentriacontane		C_31_H_64_	436	11.939	1,656,367	8.956
Octadecane		C_18_H_38_	254	12.545	842,691	4.556
Pentadecane, 2-methyl-		C_16_H_34_	226	14.531	1,946,339	10.524
Hexacosane		C_26_H_54_	366	15.235	1,156,936	6.256

**Table 9:** Bio-alkanes produced by *B. aerophilus* Camph.3 in TSB medium

Alkane name	Structure	Formula	Molecular weight	Retention time	Peak area	mg alkane/L culture
Heptane, 2,2,4,6,6-pentamethyl-		C_12_H_26_	170	4.426	1,284,688	5.810
Nonane, 4,5-dimethyl		C_11_H_24_	156	5.216	566,578	2.562
Heptadecane, 2-methyl-		C_18_H_38_	254	7.379	1,050,595	4.751
Eicosane		C_20_H_42_	282	7.733	836,152	3.782
Dodecane, 2,6,11-trimethyl-		C_15_H_32_	212	9.089	2,872,460	12.991
Heptacosane, 1-chloro-		C_27_H_55_Cl	414	9.170	489,970	2.216
Dodecane		C_12_H_26_	170	9.496	2,518,180	11.388
Tetracosane		C_24_H_50_	338	9.593	657,646	2.974
Hexadecane		C_16_H_34_	226	10.062	780,927	3.532
Pentadecane		C_15_H_32_	212	11.201	752,401	3.403
Hexacosane		C_26_H_54_	366	11.378	2,293,290	10.371
Octacosane		C_28_H_58_	394	11.939	1,562,255	7.065
Decane, 3-methyl-		C_11_H_24_	156	14.537	1,654,373	7.482
Decane, 4-methylene-		C_11_H_22_	154	14.577	967,388	4.375

The alkanes are preferred as clean fuels, because they burn cleanly and easily, releasing a lot of heat and light energy [35]. In the present study, the three studied endophytic bacteria produced a variety of alkanes as mentioned above. Many of these alkanes are used in biofuel production. Octane and decane are the main constituents of gasoline. Octane is used in internal combustion engines. Nonane, decane, undecane, tetradecane, pentadecane, and hexadecane make up the majority of diesel, kerosene, and aviation fuel. Heptadecane, octadecane, ecosane, pentacosane, hexacosane, heptacosane, octacosane, and heneicosane are the main components of lubricating oil [36].

## Conclusion

Using microorganisms is a fantastic new starting point for sustainable biofuel production. The study's findings, which were not reported previously, identified three species of bacteria as effective and environmentally benign sources for the production of different alkanes. Three endophytic bacteria were isolated from the leaves of *C. camphora* and were molecularly identified as *Bacillus atrophaeus* Camph.1 (OR343176.1), *Bacillus spizizenii* Camph.2 (OR343177.1), and *Bacillus aerophilus* Camph.3 (OR343178.1). These isolates showed great potential in producing various alkanes when grown in NB, LB, and TSB media. Numerous of the produced alkanes, such as octane, nonane, decane, undecane, tetradecane, pentadecane, and hexadecane are used in biofuel production, such as gasoline, diesel, kerosene, and aviation fuel. Therefore, these endophytic bacteria may be promising and sustainable sources for alkane biofuel production.

## Data Availability

The dataset supporting the conclusions of this article is available in the [NCBI] repository [https://www.ncbi.nlm.nih.gov/nuccore/OR343176.1/], [https://www.ncbi.nlm.nih.gov/nuccore/OR343177.1/], and [https://www.ncbi.nlm.nih.gov/nuccore/OR343178.1/].
